# Elevated serum concentrations of HE4 as a novel biomarker of disease severity and renal fibrosis in kidney disease

**DOI:** 10.18632/oncotarget.11682

**Published:** 2016-08-29

**Authors:** Jianxin Wan, Yanhong Wang, Gaorong Cai, Jianbo Liang, Caifeng Yue, Fen Wang, Junli Song, Jianfeng Wang, Min Liu, Jinmei Luo, Laisheng Li

**Affiliations:** ^1^ Department of Laboratory Medicine, The First Affiliated Hospital of Sun Yat-sen University, Guangzhou, People's Republic of China; ^2^ Department of Internal Medicine, Medical Intensive Care Unit and Division of Respiratory Diseases, Third Affiliated Hospital of Sun Yat-sen University, Guangzhou, People's Republic of China; ^3^ Department of Pathology, The First Affiliated Hospital of Sun Yat-sen University, Guangzhou, People's Republic of China; ^4^ Reproductive Medicine Center, The First Affiliated Hospital of Sun Yat-sen University, Guangzhou, People's Republic of China; ^5^ Institute of Laboratory Medicine, Guangdong Medical College, Dongguan, People's Republic of China

**Keywords:** HE4, diagnostic, renal fibrosis, kidney disease, biomarkers, Pathology Section

## Abstract

**Background:**

Human epididymis protein 4 (HE4), has recently been reported as a mediator of renal fibrosis. However, serum HE4 levels appear in a large number of patient samples with chronic kidney disease (CKD), and the relationship of these levels to disease severity and renal fibrosis is unknown.

**Methods:**

In 427 patients at different stages of CKD excluding gynecologic cancer and 173 healthy subjects, serum HE4 concentrations were tested by chemiluminescent microparticle immunoassay. Renal biopsy was performed on 259 of 427 subjects. Histological findings were evaluated using standard immunohistochemistry.

**Results:**

The levels of serum HE4 were higher in CKD patients than in healthy subjects, and higher levels were associated with more severe CKD stages. Patients with more severe renal fibrosis tended to have higher HE4 levels, and correlation analysis showed a significant correlation between HE4 and degree of renal fibrosis (*r* = 0.938, *P* < 0.0001). HE4 can be a predictor of renal fibrosis in CKD patients; the area under the receiver-operating characteristic curve (AUC-ROC) was 0.99, higher than the AUC-ROC of serum creatinine (0.89).

**Conclusion:**

Elevated levels of serum HE4 are associated with decreased kidney function, and also with an advanced stage of renal fibrosis, suggesting that HE4 may serve as a valuable clinical biomarker for renal fibrosis of CKD.

## INTRODUCTION

Renal fibrosis is the common final end point of histological manifestation of virtually all progressive kidney diseases. Most chronic renal damage, regardless of etiology, leads to renal fibrosis. Renal fibrosis is characterized by the excessive and persistent accumulation of extracellular matrix (ECM), myofibroblasts, and infiltrating inflammatory cells which are responsible for the advanced destruction of normal tissue architecture of the kidney and eventually causes progressive loss of kidney function [1]. Renal fibrosis is commonly considered irreversible, which results in glomerular sclerosis, tubular atrophy or dilation, interstitial fibrosis and rarefaction of the glomerular, as well as peritubular capillaries. Indeed, irrespective of the initial cause, after the primary occurrence, kidney tissue undergoes a series of processes, which include activation of kidney resident cells, release of pro-inflammatory cytokines, and infiltration of inflammatory monocytes/macrophages, and T cells to the damaged positions. If damage persists, glomerular or interstitial infiltrated inflammatory cells turn into activated and secret pro-fibrotic and inflammatory cytokines, reactive oxygen species, and other detrimental molecules [2]. Finally, mesangial cells, tubular epithelial cells, and fibroblasts are then activated and undergo phenotypic transition and produce many ECM proteins. Myofibroblasts are commonly regarded as the predominant effector cells that are responsible for renal fibrosis formation. Therefore, their activation is regarded as a fundamental event in the pathogenesis of renal fibrosis [3, 4]. Myofibroblasts are often indicated to represent activated or differentiated fibroblasts; the origin of myofibroblasts include bone marrow-derived fibroblasts, tubular epithelial cells, endothelial cells, pericytes, and interstitial fibroblasts [5]. However, the mechanism of myofibroblast activation and renal fibrogenesis have not been thoroughly elucidated [6]. Recently, a study revealed *HE4* as the most upregulated gene in fibrosis-associated myofibroblasts, which could be a potential biomarker for diagnosis of renal fibrosis [7].

Human epididymis secretory protein 4 (HE4, also known as WFDC2) is a N-glycosylated protein, which is encoded by a gene located on chromosome 20q12-13.1. This protein was primarily identified as a transcript exclusively expressed in the epididymis and concerned with maturation of sperm [8]. After the initial study, HE4 was also found expressed in respiratory tracts, the oral cavity, and it might play an important role in the process of innate immunity defenses and tumorigenesis of lung adenocarcinoma [9]. In addition, HE4 was reported over-expressed in ovarian cancer patients' serum even in early-stage diseases, especially in serous and endometrioid epithelial ovarian cancer [10]. HE4 has been proved to be appropriate as a serum biomarker in epithelial ovarian cancer by several study groups, and it was also approved to act as a monitoring index of recurrent or progressive disease by the United States Food and Drug Agency (FDA) in 2009 [11]. More recently, *LeBleu VS et al.* identified HE4 as a mediator of renal fibrosis, which was upregulated in human and mouse fibrotic kidneys, through its capability as a protease inhibitor to suppress the activity of multiple proteases, such as serine proteases and matrix metalloproteinases, especially by inhibiting their ability to degrade type I collagen [7]. In addition, they also found serum levels of HE4 elevated in patients with kidney fibrosis, which could become a potential serum biomarker of renal fibrosis. Interestingly, in chronic kidney disease (CKD) patients, serum HE4 concentrations were often abnormally elevated in patients, even at early stages [12]. These observations have led to the proposal that serum HE4 may serve as a new potential biomarker to assess renal fibrosis. However, the clinical diagnostic capability of serum HE4 for renal fibrosis in clinical samples remains unknown [1, 13]. Thus, the purpose of this study was to evaluate serum HE4 as a diagnostic marker of renal fibrosis in patients with kidney disease.

## RESULTS

### Clinical characteristics

Baseline characteristics of the study participants are presented in Table 1 according to the stage of renal function. Overall, the mean age of our study group was 38.6 years, and 222 of 427 were men. For the control group, mean age was 40.0 years, and 79 of 173 were men. The mean age of patients with CKD stage 2 (31.0 years) was lower than those with advanced stages of CKD. The primary etiology of CKD was as follows: chronic glomerulonephritis in 268 (62.8 %) patients, renal transplant in 55 (12.9 %), diabetic nephropathy in 48 (11.2 %), hypertension in 29 (6.8 %), nephrolithiasis in 21 (4.9 %), and other in 6 (0.2 %). Median serum creatinine and estimated glomerular filtration rate (eGFR) were 211 μmol/L and 28.9 mL/min/1.73 m^2^, respectively. Mean serum hemoglobin, uric acid, calcium, and phosphorus concentrations were 11.2 g/dL, 6.9 mg/dL, 8.5 mg/dL, and 4.1 mg/dL, respectively. The median HE4 in CKD patients was 329 (interquartile range (IQR), 146.1-750.2) pmol/L, and in the control group it was 35.4 (IQR, 30.9-42.9) pmol/L (*P* < 0.0001). The detailed median levels of serum HE4 in CKD patients and healthy controls are given in Table 2. There were no obvious differences in HE4 levels between men and women in controls and CKD patients (*P* = 0.2683, *P* = 0.3740, respectively; Table 2). HE4 served as a biomarker for the detection of ovarian cancer in women with a pelvic mass; the cut-off value of HE4 was 70 pmol/L and 140 pmol/L for premenopausal and postmenopausal women, respectively, according to the manufacturer's instructions. Therefore, we analyzed serum HE4 levels of both men and women, respectively. For men, serum HE4 levels were significantly higher in the older group (≥50 years) than in the young group (<50 years) in controls and CKD patients (*P* = 0.0011, *P* = 0.0413, respectively; Table 2). For women, serum HE4 levels were obviously higher in postmenopausal women than in premenopausal women in controls (50.0 pmol/L and 35.2 pmol/L, respectively; *P* < 0.0001). However, there was no obvious difference in HE4 levels between postmenopausal women and premenopausal women in CKD patients (360.9 pmol/L and 283.9 pmol/L, respectively; *P* = 0.0921).

**Table 1:** Clinical characteristics and laboratory tests of all study participants, CKD subgroups based on their renal function

Variables	Controls (*n*=173)	Total (*n*=427)	CKD2 (*n*=109)	CKD3 (*n*=99)	CKD4 (*n*=79)	CKD5 (*n*=140)
Age (y)	40.0±11.3	38.6±14.3	31.0±10.9	39.8±13.5	42.6±15.1	41.3±14.7
Gender (M/F), n, (n/n)	173 (79/94)	427 (222/205)	109 (61/48)	99 (56/43)	79 (33/46)	140 (72/68)
BMI (kg/m^2^)	-	23.7±3.5	23.2±3.6	24.1±4.0	24.0±3.7	23.5±2.9
SBP (mm Hg)	-	133.1±17.7	126.3±15.2	131.7±16.7	133.8±17.9	139.1±20.2
DBP (mm Hg)	-	78.4±13.4	77.9±10.3	78.5±14.4	77.2±16.8	79.3±13.1
Primary disease						
Diabetes	-	48 (11.2 %)	15 (3.5 %)	11 (2.6 %)	7 (1.6 %)	15 (3.5 %)
Hypertension	-	29 (6.8 %)	15 (3.5 %)	6 (1.4 %)	3 (0.7 %)	5 (1.2 %)
Glomerulonephritis	-	268 (62.8 %)	47 (11.0 %)	58 (13.6 %)	55 (12.9 %)	108 (25.3 %)
Nephrolithiasis	-	21 (4.9 %)	5 (1.2 %)	8 (1.9 %)	3 (0.7 %)	5 (1.2 %)
Transplant	-	55 (12.9 %)	26 (6.1 %)	14 (3.3 %)	11 (2.6 %)	4 (0.9 %)
Other	-	6 (0.2 %)	1 (0.5 %)	2 (11.2 %)	0 (0 %)	3 (0.7 %)
Laboratory tests						
Scr (μmol/L)	65.0 (60.0-72.5)	211.0 (130.0-612.5)*	111.0 (95.0-124.0)*	158 (142-188.5)*	256 (217.5-293.5)*	860.5 (622-1078.3)*
eGFR (mL/min/1.73 m^2^)	-	28.9 (8-60.5)	72 (67-78)	40 (34-48)	21.5 (18.5-26)	5.5 (3.8-8)
Hemoglobin (g/dL)	-	11.2±2.2	13.1±1.6	11.4±2.3	10.2±2.6	10.1±2.4
Uric acid (mg/dL)	-	6.9±2.4	5.6±1.7	6.9±2.2	7.4±2.6	7.5±3.1
Calcium (mg/dL)	-	8.5±0.8	8.8±0.9	8.6±0.7	8.3±0.7	8.2±0.8
Phosphorus (mg/dL)	-	4.1±1.1	3.7±0.6	3.9±0.7	4.2±1.4	4.6±1.7
HE4 (pmol/L)	35.4 (30.9-42.9)	329 (146.1-750.2)*	90.6 (59.3-141.5)*	201.7 (156.5-303)*	371 (305.1-494.5)*	1146.3 (739.7-1658)*

Data are presented as n (%) or means ± SD for normally distributed continuous variables, median and interquartile range for continuous variables that are not normally distributed. CKD stage was defined as followed: CKD2, eGFR of 60-89 mL/min/1.73 m^2^; CKD3, eGFR of 30-59 mL/min/1.73 m^2^; CKD4, eGFR 15-29 mL/min/1.73 m^2^; and CKD5, eGFR<15 mL/min/1.73 m^2^.

* Compared to corresponding normal control, Scr or HE4 levels showed significant difference (all *P*<0.01). Mann-Whitney U-test was used.

**Table 2:** Association between HE4 levels and characteristical variables in CKD patients/normal controls

	Number	HE4 (pmol/L)	*P*values
Combined			
Controls	173	35.4 (30.9-42.9)	<0.0001
CKD	427	329.0 (146.1-750.2)	
Controls			
Men	79	35.7 (30.6-41.3)	0.2683
Women	94	37.8 (32.8-43.9)	
CKD			
Men	222	346.8 (152.0-972.2)	0.3740
Women	205	325.0 (137.3-626.2)	
Men			
Controls			
<50 (Y)	62	34.1 (30.4-38.6)	0.0011
≥50 (Y)	17	49.5 (41.3-68.3)	
CKD			
<50 (Y)	167	294.0 (141.8-747.4)	0.0413
≥50 (Y)	55	579.0 (213.0-1393.8)	
Women			
Controls			
Premenopausal	71	35.2 (31.3-39.7)	<0.0001
Postmenopausal	23	50.0 (41.0-59.8)	
*CKD*			
Premenopausal	164	283.9 (121.0-622.0)	0.0921
Postmenopausal	41	360.9 (297.7-634.3)	

The association between HE4 levels and characteristical variables were analyzed by Mann-Whitney U-test.

### Relationship between serum HE4 level and CKD stages

**Figure 1:**
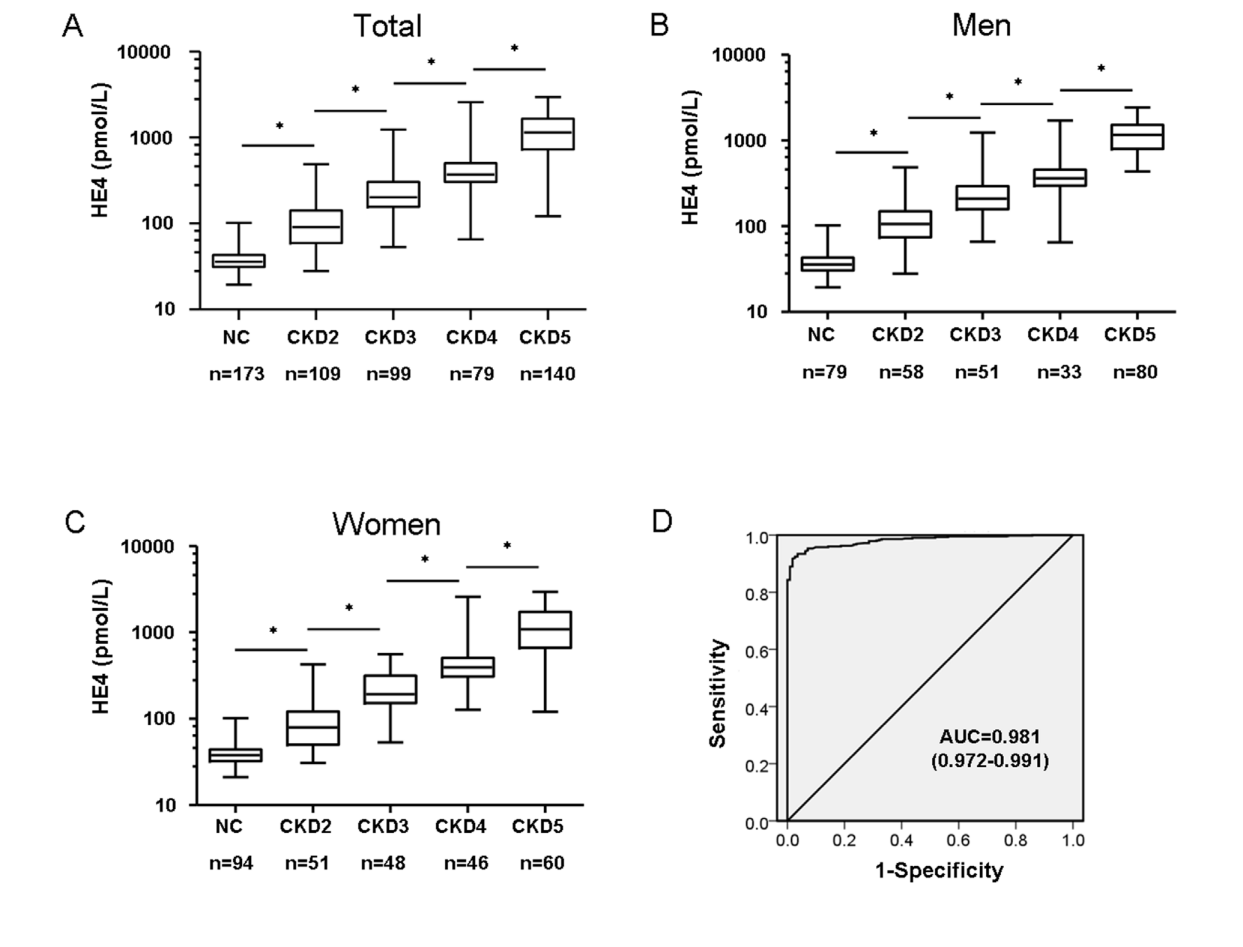
Serum HE4 is strongly associated with progression of CKD in patients **A.** Differential levels of serum HE4 in patients with CKD or NC (normal healthy control) group. Results indicated that serum HE4 levels were elevated in patients with CKD, and increased with a higher stage of CKD (*P < 0.0001). **B.** Differential levels of serum HE4 for male patients. Serum HE4 levels are elevated in male patients with CKD (*P < 0.0001). **C.** Differential levels of serum HE4 for female patients. Serum HE4 levels are elevated in female patients with CKD (*P < 0.0001). **D.** Receiver-operating characteristic curve analysis displaying the diagnostic power in predicting disease severity of CKD in patients by serum HE4 levels (area under the curve, AUC: 0.981).

Serum HE4 concentrations were higher (*P* for trend <0.0001) in more advanced CKD stages (Figure 1A, 1B, 1C) and strongly associated with several CKD risk factors at baseline. Therefore, Spearman's correlation analysis was used to determine the correlation between serum HE4 and creatinine, and also eGFR (Table 3). We observed a strong positive correlation between HE4 and creatinine (*r* = 0.9018; *P* < 0.0001), whereas a modest inverse correlation between HE4 and eGFR (*r* = −0.7077; *P* < 0.0001) was shown. Meanwhile, correlation analyses also demonstrated that serum HE4 levels positively correlated with age (*r* = 0.2041; *P* < 0.0001), systolic blood pressure (SBP) (*r* = 0.1493; *P* = 0. 0021), uric acid levels (*r* = 0.3023; *P* < 0.0001), and phosphorus levels (*r* = 0.2050; *P* < 0.0001), whereas serum HE4 was inversely correlated with hemoglobin (*r* = −0.3710; *P* < 0.0001) and calcium levels (*r* = −0.2821; *P* < 0.0001; Table 3). The predictive power of serum HE4 for detecting severity of CKD patients was evaluated by ROC analysis. The area under the receiver-operating characteristic curve (AUC-ROC) of serum HE4 predictive ability was 0.981 (95% CI, 0.972-0.991; Figure 1D).

**Table 3:** Spearman correlation coefficients between HE4 and other variables

	Age	SBP	Scr	eGFR	Hb	UA	Ca	Phosphorus
HE4								
r	0.2041	0.1493	0.9018	−0.7077	−0.3710	0.3023	−0.2821	0.2050
P	<0.0001	0.0021	<0.0001	<0.0001	<0.0001	<0.0001	<0.0001	<0.0001
Age								
r	1	0.0793	0.1598	−0.2732	−0.3251	0.1336	−0.2275	0.0272
P	-	0.2614	<0.0001	<0.0001	<0.0001	0.0311	<0.0001	0.5532
SBP								
r		1	0.2949	−0.3258	−0.1381	0.1523	−0.1222	0.2317
P		-	<0.0001	<0.0001	0.0271	0.0224	0.0614	<0.0001
Scr								
r			1	−0.9763	−0.4720	0.2785	−0.3172	0.2680
P			-	<0.0001	<0.0001	<0.0001	<0.0001	<0.0001
eGFR								
r				1	0.5128	−0.2574	0.2741	0.2482
P				-	<0.0001	<0.0001	<0.0001	<0.0001
Hb								
r					1	−0.4362	0.4830	−0.3011
P					-	<0.0001	<0.0001	<0.0001
UA								
r						1	−0.2672	0.2314
P						-	<0.0001	<0.0001
Ca								
r							1	−0.3671
P							-	<0.0001
Phosphorus								
r								1
P								-

Correlations between two variables were performed by Spearman's correlation analysis.

### Association between serum HE4 levels and renal fibrosis

**Figure 2:**
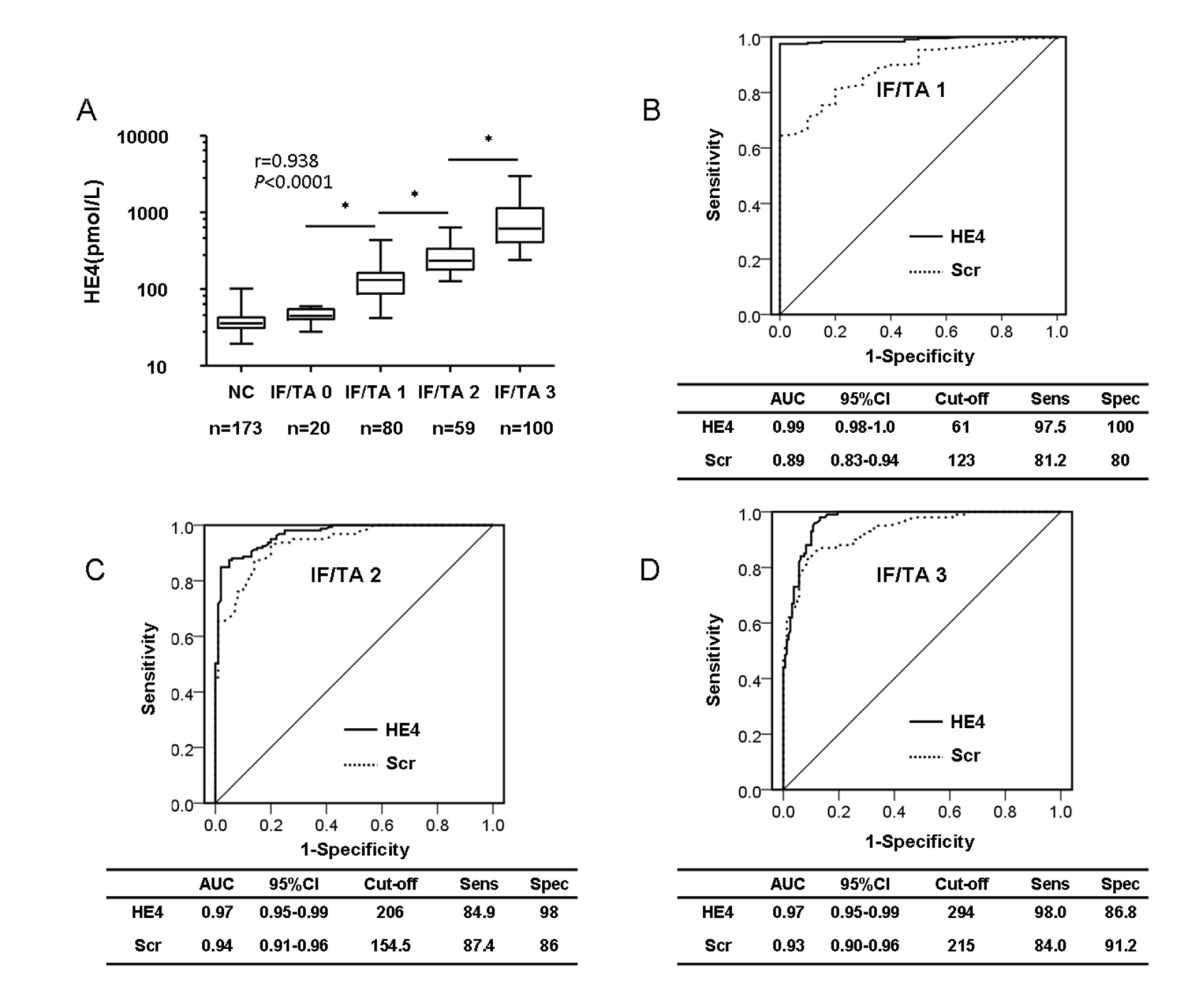
Serum HE4 level is a potential biomarker of renal fibrosis **A.** Serum HE4 levels are elevated in CKD patients with renal fibrosis, and increased with IF/TA grade (*P < 0.0001). **B.** ROC analysis displaying the diagnostic power of serum HE4 in predicting renal fibrosis in CKD patients (IF/TA 1, AUC: 0.990). **C.** ROC analysis displaying the diagnostic power of serum HE4 in predicting modest renal fibrosis in CKD patients (IF/TA 2, AUC: 0.968). **D.** ROC analysis displaying the diagnostic power of serum HE4 in predicting advanced renal fibrosis in CKD patients (IF/TA 3, AUC: 0.969).

To further determine whether serum HE4 levels were associated with renal fibrosis, or even serve as a diagnostic biomarker of renal fibrosis, 259 patients who had received renal biopsies were categorized into different groups according to each patient's renal fibrosis score, as detailed in Table 4. Our results indicated that patients with more advanced renal fibrosis tended to be older and had a higher SBP, serum creatinine, uric acid, and HE4 levels, and lower eGFR (*P* for trend <0.0001, for all). We then analyzed the correlation between renal fibrosis and serum HE4, as shown in Figure 2A; a significantly positive correlation was observed when renal fibrosis was plotted against HE4 levels (two-tailed Spearman's correlation, *r* = 0.938, *P* < 0.0001), and serum HE4 levels were obviously elevated with IF/TA fibrosis grade (serum HE4 levels of normal control, IF/TA 0, 1, 2 and 3: 35.9, 45.0, 131.5, 234.0, and 615.0, pmol/L, *P* < 0.0001). The predictive power of serum HE4 for distinguishing renal fibrosis from CKD patients was evaluated by ROC analysis. The AUC-ROC of serum HE4 predictive ability was 0.990 (95% CI, 0.981-0.999) with a sensitivity and specificity at 97.5 % and 100.0 %, respectively (cut-off value was 61 pmol/L), which was higher than that of serum creatinine, which was 0.89 (95% CI, 0.83-0.94) with a sensitivity and specificity at 81.2 % and 80.0 %, respectively (Figure 2B). In discriminating modest renal fibrosis (IF/TA 2+3) from no or mild fibrosis in CKD patients, the AUC-ROC of serum HE4 predictive ability was 0.968 (95% CI, 0.950-0.986) with a sensitivity and specificity at 84.9 % and 98.0 %, respectively (cut-off value was 206 pmol/L), which was also higher than the AUC-ROC of serum creatinine AUC (0.94, 95% CI, 0.91-0.96) with a sensitivity and specificity at 87.4 % and 86.0 %, respectively, (Figure 2C). Then, in differentiating advanced-stage renal fibrosis (IF/TA 3), the AUC-ROC of serum HE4 was 0.97 (95% CI, 0.95-0.99) with a sensitivity and specificity at 98.0 and 86.8, respectively (cut-off value was 294 pmol/L), higher than that of serum creatinine (0.93, 95% CI, 0.90-0.96) with a sensitivity and specificity at 84.0 % and 91.2 %, respectively (Figure 2D). Table 5 shows the net reclassification improvement (NRI) analysis for serum HE4. Overall, 15 patients with renal fibrosis moved up after being reclassified; 17 patients without renal fibrosis were reclassified false positive. Therefore, the NRI was 0.91 (*P* < 0.01), indicating that HE4 improves the prediction of the risk of renal fibrosis 91%, compared with creatinine, and was statistically significant.

**Table 4:** Clinical characteristics and laboratory tests of study participants according to renal fibrosis score

Variable	IF/TA 0	IF/TA 1	IF/TA 2	IF/TA 3	*P* value
n	20	80	59	100	-
Age (y)	30.6±11.5	31.9±11.6	36.4±13.2	45.3±14.2	<0.0001
Gender (M/F), n/n	9/11	58/22	36/23	54/46	-
BMI (kg/m^2^)	23.7±3.6	23.9±3.9	24.1±3.6	23.8±3.1	0.6271
SBP (mm Hg)	123.1±16.3	128.9±17.6	134.5±16.5	138.9±19.4	<0.0001
DBP (mm Hg)	77.1±11.3	78.2±15.3	78.4±14.2	78.9±14.8	0.4874
Primary disease					
Diabetes	4 (1.5 %)	6 (2.3 %)	4 (1.5 %)	8 (3.1 %)	-
Hypertension	0 (0 %)	6 (2.3 %)	2 (0.8 %)	3 (1.2 %)	-
Glomerulonephritis	6 (2.3 %)	53 (20.5 %)	43 (16.6 %)	75 (29.0 %)	-
Nephrolithiasis	1 (0.4 %)	1 (0.4 %)	1 (0.4 %)	3 (1.2 %)	-
Transplant	8 (3.1 %)	14 (5.4 %)	7 (2.7 %)	10 (3.9 %)	-
Other	0 (0 %)	1 (0.4 %)	2 (0.8 %)	1 (0.4 %)	-
Laboratory tests					
Scr (μmol/L)	97.5 (86.8-122.0)	123.0 (101.0-142.0)	195.0 (153.5-213.0)	412.0 (249.3-847.0)	<0.0001
eGFR (mL/min/1.73 m^2^)	77.0 (69.3-83.0)	65.5 (55.0-75.0)	33.8 (28.0-40.4)	12.4 (6.0-22.0)	<0.0001
Hemoglobin (g/dL)	13.6±1.4	11.9±1.9	10.1±2.1	9.8±1.8	<0.0001
Uric acid (mg/dL)	5.1±1.4	6.2±1.9	7.0±2.1	7.7±2.6	<0.0001
Calcium (mg/dL)	8.9±0.7	8.7±0.6	8.4±0.7	8.3±0.8	0.0740
Phosphorus (mg/dL)	3.6±0.5	3.8±0.6	4.0±1.3	4.4±1.6	0.0023
HE4 (pmol/L)	45.0 (40.7-54.9)	131.5 (87.9-160.4)	234.0 (180.0-329.8)	615.0 (412.5-1108.0)	<0.0001

Data are presented as n (%) or means ± SD for normally distributed continuous variables, median and interquartile range for continuous variables that are not normally distributed.

The variables between different groups were analyzed by Kruskal-Wallis test.

## DISCUSSION

In this study, we showed that serum HE4 levels were significantly elevated in patients with CKD and were associated with eGFR. Meanwhile, we also found that serum HE4 levels were obviously elevated in advanced CKD stages, suggesting that HE4 can be a novel biomarker for predicting the severity of CKD. In addition, we demonstrated that serum HE4 levels were significantly positively associated with renal fibrosis in CKD patients. HE4 elevation obviously increased with advanced renal fibrosis stage, and ROC analysis showed HE4 as a suitable biomarker, which was more sensitive than serum creatinine for the diagnosis of renal fibrosis in CKD patients. We also calculated the cut-off value for HE4 in CKD patients with renal fibrosis.

**Table 5:** NRI analysis for HE4

NRI analysis for HE4							
Model without HE4	Model with HE4				Reclassified		
	<50%	50-80%	>80%	Total	Increased risk	Decreased risk	Net correctly reclassified
Patients with renal fibrosis							
<50%	0 (0.00)	1 (100.00)	0 (0.00)	1	20	5	15
50-80%	3 (13.04)	1 (4.35)	19 (82.61)	23			
>80%	2 (0.93)	0 (0.00)	213 (99.07)	215			
Total	5	2	232	239			
Patients without renal fibrosis							
<50%	1 (100.00)	0 (0.00)	0 (0.00)	1	0	17	−17
50-80%	9 (81.81)	2 (18.18)	0 (0.00)	11			
>80%	5 (62.5)	3 (37.5)	0 (0.00)	8			
Total	15	5	0	20			
*Data are frequency (row percentage). NRI=0.91 (*P*<0.01)							

HE4 has recently been highlighted as a mediator and biomarker in kidney fibrosis [13]. HE4 and its downstream targets, Prss35 and Prss23 levels, were elevated in human fibrotic kidneys [7]. In addition to upregulated expression, HE4 exerts a putative serine protease inhibitor activity, through inhibiting Prss35 and Prss23 serine protease activities and preventing the degradation of type I collagen. In addition, serum HE4 levels were elevated in CKD patients with renal fibrosis suggesting that HE4 could serve as a biomarker for predicting renal fibrosis. However, these preliminary findings were obtained from small-scale patient analyses (11 patients with CKD with five healthy controls), and as yet no study has reported on these. To the best of our knowledge, our work is the first large-scale clinical case study to demonstrate that high levels of serum HE4 are associated with CKD and renal fibrosis in patients.

HE4 is a functionally diverse 10- to 25-kDa member of the Whey Acidic Proteins (WAP) four-disulfide core domain 2 (WFDC2) family of WAP, a group functionally characterized by the WAP domain-containing family displaying proteinase inhibitor function [14]. Most notably, HE4 together with other proteins of this family, elafin, PS20, and SLPI, have been identified as potential molecular biomarkers for cancer [15]. HE4 has been studied extensively, especially for ovarian cancer, for which HE4 displays a tumor located, up-regulated, and secreted into blood plasma, making it a potential serum biomarker for ovarian cancer and endometrial cancer [10, 16, 17]. *Li et al.* showed that HE4 had better specificity than CA125 in discriminating diagnosis of malignant from benign gynecological diseases in a southern China population [18]. Recently, serum HE4 has been identified as a diagnostic and prognostic biomarker for lung cancer [19, 20]. In addition to cancer, HE4 has also been found to be associated with heart failure severity and outcome, and could serve as a powerful and independent prognostic biomarker for heart failure outcome [21]. *Piek A et al.* confirmed elevated HE4 levels in patients with chronic heart failure, the levels of which correlated with heart failure severity, NT-proBNP levels, and renal function [22]. Considering the strong heart-kidney function association, this work implied serum HE4 could be a biomarker for renal function evaluation, which was consistent with our study.

This study obviously demonstrated that serum HE4 levels were positively associated with creatinine in patients with CKD. Our findings are consistent with the results of a recent report by *Nagy et al.* [12]. They reported that serum HE4 levels were significantly elevated in 113 female patients with CKD compared with 68 normal healthy controls, and obviously increased in patients with advanced CKD. Moreover, serum HE4 levels were obviously correlated with serum creatinine levels. Our study extended these findings by showing significant associations with elevated serum HE4 levels and the loss of renal function and decreased eGFR in CKD patients. Taken together, these findings suggest that serum HE4 levels were elevated as CKD became severe and that they could serve as a valuable biomarker in patients with risk of CKD progression.

Renal biopsy is usually necessary to clarify a diagnosis, especially in cases of glomerular disease [23]. Despite the fact that biopsy is generally acknowledged as the gold standard of diagnostic methods, it is accompanied by risks, including hemorrhage, pain, and even death in a few patients. Until now, serum creatinine and urine protein are two of the most commonly used serum diagnostic biomarkers for renal fibrosis, but they are still not as effective as biopsy. *Psihoqios NG et al.* used NMR-based urinary metabolome analysis to evaluate the severity of renal damage and to possibly reflect kidney function. However, this technique requires very expensive instruments and professional experts, and will be performed infrequently at most hospitals, especially in a general hospital [24]. Other studies also reported using urine fatty acid-binding protein (FABP) or nucleic acids as potential markers of fibrosis [25, 26]. However, these biomarkers were obtained from clinical analysis, and there was no authentic evidence mechanism proving them to be associated with renal fibrosis. Transforming growth factor-β1 (TGF-β1) as a pro-fibrotic cytokine was also reported to play a crucial role in renal fibrosis, which can be tested in both serum and urine. *Suthanthiran et al.* reported that serum TGF-β1 levels had a positive association with several risk factors for CKD progression in African-American patients, and suggested that serum TGF-β1 can serve as a reliable biomarker for CKD progression [27]. Recently, several groups demonstrated that microRNAs play important roles in the pathogenesis of different kidney diseases, and some serum or urine microRNAs could be suggested as useful biomarkers of renal fibrosis in patients with CKD, such as miR-21, miR-29c, miR-25, miR-148b, and miR-150 [21, 28]. However, microRNA expression levels vary from individual to individual and measurement of microRNAs is a highly qualified and expensive work, which has a long way to go toward clinical diagnosis application. In this study, we performed an HE4 test by chemiluminescent microparticle immunoassays (CMIA) using the fully automated ARCHITECT instrument. The whole process is simple and fast, and the results demonstrated serum HE4 to be significantly associated with renal fibrosis in CKD patients, and ROC analysis demonstrated that serum HE4 is more suitable as an eligible biomarker for distinguishing renal fibrosis from CKD patients than serum creatinine (AUC-ROC of HE4 *vs*. creatinine was 0.99 *vs*. 0.89, respectively). Calculated NRI also demonstrated that HE4 significantly improves the prediction risk of renal fibrosis, compared with creatinine (NRI = 0.91, *P* < 0.01).

There are several limitations to this study. First, we measured serum HE4 only by CMIA on the fully automated ARCHITECT instrument and results were not confirmed by western blot. *LeBleu VS et al.* tested serum HE4 by both western blot and enzyme-linked immunosorbent assay methods, and found serum HE4 levels to be elevated in patients with CKD [7]. Meanwhile, *Nagy et al.* used the same kit that we used to test serum HE4 in CKD patients [12]. Second, it was a single-center design with relatively more of the end-stage patient population. A large multiracial, multicenter study with more early-stage CKD patients, such as stage 1, is required to determine the importance of HE4 in renal fibrosis in patients with CKD. Third, study subjects were recruited from August 2013 to July 2015, with no survival analysis, which requires follow-up data, to determine the prognosis. Fourth, renal fibrosis pathology results were acquired from kidney biopsy, which was performed in some of the patients; therefore, results might not be representative of all the study patients. In addition, TGF-β1 was not tested in our study and we could not compare the usefulness of serum HE4 with existing markers.

In conclusion, we demonstrated that elevated levels of serum HE4 at the time of biopsy were associated with decreased kidney function, and that HE4 elevation levels obviously increased with advanced renal fibrosis stage in patients with CKD, suggesting that HE4 may serve as a valuable clinical biomarker for renal fibrosis of CKD.

## MATERIALS AND METHODS

### Patient population and study design

The study group comprised a cohort of patients with CKD recruited in the First Affiliated Hospital of Sun Yat-sen University from August 2013 to July 2015. CKD patients were selected according to National Kidney Foundation-Kidney Disease Outcomes Quality Initiative (NKF KDOQI) criteria [29]. Study participants were inpatients at the department of nephrology of our hospital due to persistent proteinuria/microhematuria, low GFR, continued fall in eGFR, or dialysis. Of this study, a total of 427 eligible participants were recruited if they were older than 18 years and had no proof of acute kidney injury. The normal healthy controls group consisted of 173 age-matched healthy subjects with normal renal function (eGFR > 90 mL/min/1.73 m^2^) and ovarian function, and no HBV carriers. The ethics committee of the First Affiliated Hospital of Sun Yat-sen University approved the study according to Declaration of Helsinki guidelines. All subjects enrolled in this study gave written informed consent.

### Tissue processing

In this study, renal biopsies were performed on 259 of 427 subjects; pathologic samples were processed using H&E staining to evaluate the glomerular, renal tubular, and interstitial conditions through conventional histological procedures in the department of pathology of our hospital. Renal fibrosis severity was evaluated according to histological criteria (interstitial fibrosis (ci) and tubular atrophy (ct) scores) defining Interstitial Fibrosis/Tubular Atrophy (IF/TA, 2007 Banff classification) [30]. Briefly, the grading of renal fibrosis was based on the percentage of cortical parenchymal involved, as follows: IF/TA 0, interstitial fibrosis ≤ 5% cortical area; IF/TA 1: 6-25%; IF/TA 2: 26-50%; IF/TA 3: >50%.

### Data collection and laboratory tests

Patient demographics and clinical data were recorded, including age, sex, primary kidney disease, body mass index, blood pressure (SBP and DBP, respectively), and medication history. For laboratory tests, blood samples were drawn in the morning after an overnight fast of at least 8 hours. Blood and spot urine sample collection were executed between 7 AM and 10 AM. Blood samples were centrifuged immediately and tested within 2 h in the clinical laboratory of our hospital. HE4 was tested by ARCHITECT HE4 assay (Abbott Diagnostics, Abbott Park, IL) according to the manufacturer's instructions.

### Investigation of study outcomes

First, we carried out a cross-sectional study to investigate the relationship between serum HE4 levels and eGFR. All participants were classified into four subgroups according to the criteria for CKD: CKD2, eGFR of 60-89 mL/min/1.73 m^2^; CKD3, eGFR of 30-59 mL/min/ 1.73 m^2^; CKD4, eGFR of 15-29 mL/min/1.73 m^2^; and CKD5, eGFR < 15 mL/min/1.73 m^2^ (www.renal.org). Then, we compared serum HE4 levels according to CKD stages and analyzed the correlation between HE4 levels and eGFR. We also investigated the relationship between HE4 levels and renal fibrosis.

### Statistical analyses

All variables with normal distributions were shown as the mean ± standard deviation. Comparisons between variables were done by *t* test or one-way analysis of variance. To assess differences between groups, we used the Mann-Whitney U test and Kruskal-Wallis test. Spearman's correlation analysis was used to determine the correlation between two variables. The diagnostic performance of serum HE4 and creatinine for renal fibrosis was determined using ROC curves, and sensitivity, specificity, AUC, and the 95% confidence intervals were calculated. To evaluate the added predictive ability of HE4 for renal fibrosis, the NRI was assessed as described in *Pencina MJ et al.* [23]. For CKD patients with renal fibrosis, risk classification is assumed improved if the subject moves to a higher risk category with the addition of HE4, and worsened if the subject moves to a lower one. For CKD patients without renal fibrosis, the reverse is correct. In renal fibrosis patients, the difference in the proportion of subjects moving up and down a category was set up, and in patients without renal fibrosis, the proportion of subjects moving down minus the proportion moving up a category was set up. The sum of these two values was the NRI. In all cases, two-sided *P* < 0.05 was considered statistically significant. Data analyses were performed using SPSS Version 18.0 (SPSS Inc., Chicago, IL, USA).
